# Hyperparathyroidism Jaw-Tumor Syndrome: A Case Report From a Radiological View

**DOI:** 10.7759/cureus.28329

**Published:** 2022-08-23

**Authors:** Muhammad Mehraiz Khan, Huda Fazli, Touseef Bilal Khan, Pooya M Tehrany, Niempa Bacani

**Affiliations:** 1 Radiology, Institute of Nuclear Medicine & Oncology Lahore, Lahore, PAK; 2 Radiology, Services Hospital, Lahore, PAK; 3 Radiology, Mayo Hospital, Lahore, PAK; 4 Orthopedics, National University of Malaysia, Kuala Lampur, MYS; 5 Surgery, Avalon University School of Medicine, Arizona, USA

**Keywords:** mri, syndrome, cdc73 gene, jaw tumors, hyperparathyroidism

## Abstract

Limited formal guidelines, scarcity of cases, and variable manifestation forms all contribute to the challenges of diagnosing hyperparathyroidism-jaw tumor (HPT-JT) syndrome. This condition characterized by parathyroid tumors, fibro-osseous jaw tumors, and renal and gynecological pathologies results in significant morbidity, restricted functionality, and malignancy risk. Genetic testing is the gold standard investigation to evaluate for CDC73 mutations, that cause HPT-JT syndrome. Genetic testing for CDC73 mutations should be encouraged among family members of affected individuals. Surgery is the mainstay of treatment for many of the encountered pathologic entities.

We report a 42-year-old female with a history of infertility and right subtrochanteric femoral fracture secondary to osteoporosis. The patient was suspected to have primary hyperparathyroidism secondary to parathyroid adenomas that were later biochemically and scintigraphically proved with subsequent partial parathyroidectomy.

One and a half years following the initial presentation, the patient developed gradual swelling of the lower face with regional osseous involvement in addition to the clinical and radiological picture of recurrent parathyroid adenoma. We present this rare diagnosis of HPT-JT syndrome to promote awareness among physicians regarding this essential differential diagnosis. A low threshold for genetic testing and a high index of suspicion for HPT-JT syndrome must be kept in cases of patients presenting with high parathyroid hormone levels and masses. The screening must extend to all the family members as well. With this approach, the high morbidity, facial disfigurement, and significant malignancy risk can be lowered in the affected individuals improving their life expectancy.

## Introduction

Limited formal guidelines, scarcity of cases, and variable manifestation in forms all contribute to the challenges of diagnosing hyperparathyroidism-jaw tumor (HPT-JT) syndrome [1]. It is a rare diagnostic entity, an autosomal dominant familial pathology with incomplete penetrance, encompassing multiple pleiotropic phenomena including primary hyperparathyroidism secondary to parathyroid adenomas or carcinomas, fibro-osseous jaw tumors, renal and uterine pathologies [2]. Germline mutations in cell division cycle 73 (CDC 73) gene encoding parafibromin are the main culprit in the development of HPT-JT syndrome secondary to halted anti-proliferative activity. Primary hyperparathyroidism is the main pathology encountered in patients with HPT-JT syndrome with 80% uniglandular involvement and a high prevalence of atypical parathyroid adenoma [3]. Up to 20% of cases may exhibit parathyroid carcinoma [4]. Surgery is the mainstay of treatment for primary hyperparathyroidism with more advanced limited approaches reducing the risk of major post-surgical complications of permanent hypoparathyroidism. Ossifying fibromas of the maxilla or mandible are rare presentations of HPT-JT syndrome, requiring surgical correction to lessen the resulting functional limitations. Being the second most common feature of HPT-JT syndrome, uterine involvement can be benign or malignant and often presents as endometrial hyperplasia, leiomyomas, adenomyosis, adenosarcomas, or multiple adenomyomatous polyps. Uterine pathologies are seen in more than half of the CDC 73-gene carrier women. Genetic testing is the gold standard for investigation and should be performed on all the family members of HPT-JTS-affected individuals and in young patients with primary hyperparathyroidism that are candidates for surgery [4]. The rarity of HPT-JT syndrome makes its true prevalence difficult to determine. However, medical literature reports an estimated 200 cases of HPT-JT syndrome since the condition was first diagnosed in 1958 [5].

## Case presentation

A 42-year-old Sub-continental female, a known case of primary hyperparathyroidism since 2017, presented in ER with a primary complaint of acutely worsening shortness of breath. She gave a history of developing a lower facial diffuse swelling, which gradually increased in size over a period of 10 to 12 months. The swelling had restricted her jaw movements. Gradually the swelling had extended to involve the maxillary arch superiorly and anterior neck inferiorly, resulting in difficulty in eating solid foods, speech, and breathing. She also gave a history of rapid weight loss over the past six months. In her past surgical history, she had a partial parathyroidectomy done in 2017. She also had a history of right femoral fracture, for which she had an internal fixation done.

**Figure 1:**
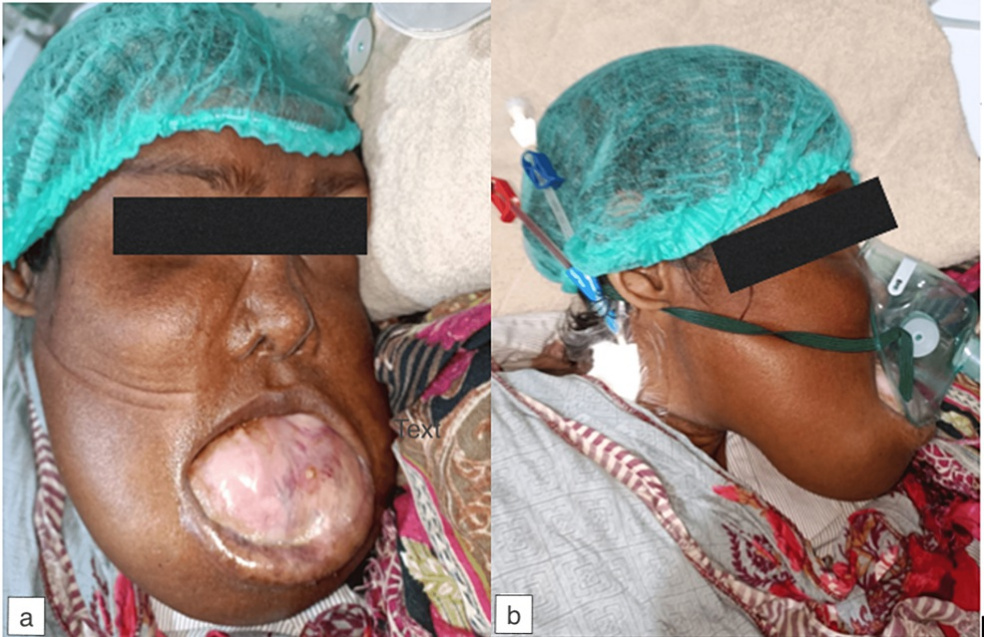
(a, b) Significant facial disfigurement caused by jaw masses.

Her saturation (SaO_2_) at presentation in ER was 88% at room air, which improved significantly when an oxygen mask was applied. The remaining vitals were unremarkable. On physical examination, a middle-aged female was seen lying in bed, not properly responding to all of the commands. Diffuse swelling of bilateral mastoid, buccal, and infraorbital, as well as mental and oral regions, was noted. There was resultant stenosis of the airway (Figures 1a, 1b). No nodal masses were appreciated on palpation. The rest of the general exam and systemic review was unremarkable.

The biochemical profile revealed raised levels of serum parathyroid hormone (PTH), serum calcium, and serum alkaline phosphatase with the values of 338 pg/mL (normal range 10-55 pg/mL), 14 mg/dL (normal range 8.6-10.3 mg/dL) and 169 IU/L (normal range 44-147 IU/L), respectively. Serum phosphorus level was reduced up to 2.2 mg/dL (normal range 3.4 to 4.5 mg/dL). Renal function tests revealed increased serum blood urea nitrogen (BUN) and creatinine levels, indicating worsening renal function.

**Figure 2:**
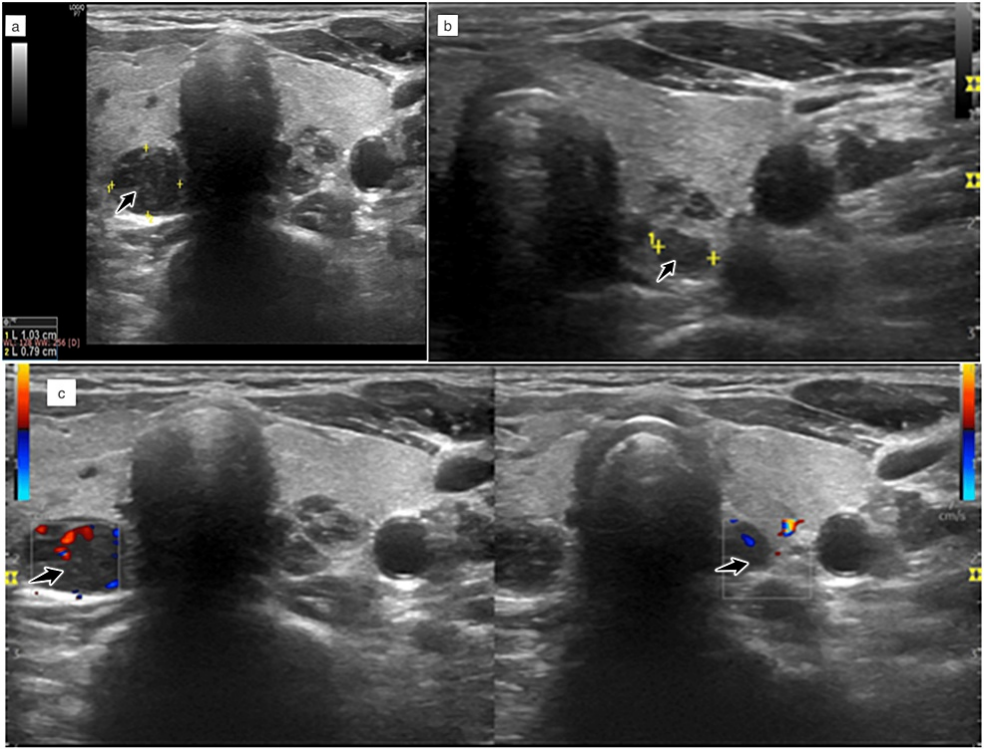
(a-c) Greyscale and color Doppler neck ultrasound (transverse views). Hypoechoic lesions on the posterior aspect of both lobes of thyroid with vascularity (marked with arrow).

A bedside neck ultrasound was performed which revealed two well-defined hypoechoic lesions, each posterior to the lobes of the thyroid gland with significant vascularity on color Doppler evaluation (Figures 2a-2c).

**Figure 3:**
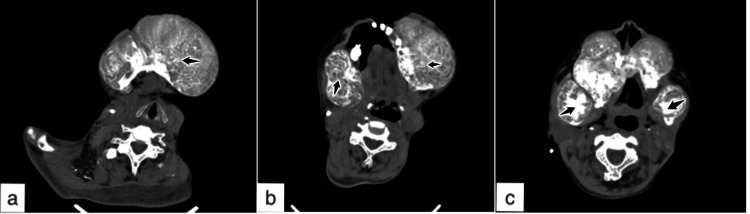
Contrast-enhanced CT face and neck (axial views). (a-c) Expansile lesions involving mandible and maxilla with internal calcified component.

A computed tomography (CT) scan was done in ER to assess the facial swelling, revealing the swelling to be of bony origin. Multiple large, well-circumscribed, sclerotic, expansile lesions involving the mandible and maxilla diffusely were seen, with extensive internal calcific components (Figures 3a-3c). No cortical break was seen. The rest of the visualized bones revealed innumerable punctate hypodense well-defined areas. No significant regional lymphadenopathy was noted. The rest of the scan was unremarkable. Imaging differentials included ossifying fibromas and fibrous dysplasia.

**Figure 4:**
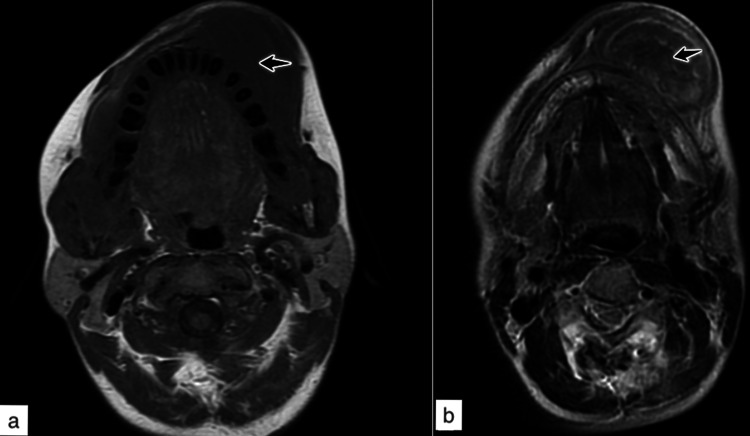
MRI face and neck (axial views) – (a) T1W and (b) T2W. Intermediate to low signal intensity multiple variable sized lesions involving maxilla and mandible.

**Figure 5:**
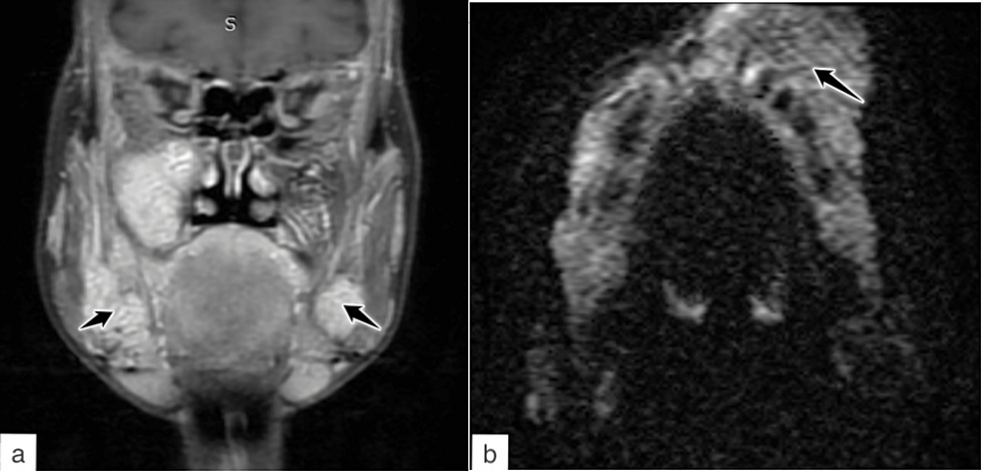
MRI face and neck – (a) coronal T1W post-contrast and (b) DWI. The lesions show post-contrast enhancement and restricted diffusion.

A subsequent magnetic resonance imaging (MRI) for anatomical evaluation and extension of jaw masses (Figures 4a, 4b, 5a, 5b) revealed multiple, bilateral, variable-sized, partially ossified soft tissue intensity mass lesions in mandible and maxilla deforming the face and partially obliterating the oral cavity. Focal low signal intensity areas are noted within the lesions on T1W and T2W sequences reflecting their calcified osseous component. The lesions showed marked post-contrast enhancement and significantly restricted diffusion. No regional lymphadenopathy was seen.

After correlating clinical, biochemical, and radiological pictures, a provisional diagnosis of HPT-JT syndrome was made. Five years before this presentation, she had presented in the Orthopedic department with a right femoral sub-trochanteric fracture which was internally fixed. She also had complain of constipation and mild weight loss at that time. Labs were in favor of primary hyperparathyroidism. A sestamibi scan was performed which showed increased uptake in the right superior parathyroid gland. DEXA scan also revealed osteoporosis. USG abdomen at that time revealed two renal calculi in the right kidney and a few cortical cysts in bilateral kidneys. Her right supra parathyroidectomy was performed and she was discharged after an uneventful postoperative recovery period.

Keeping in view the history of primary hyperparathyroidism and radiological imaging supporting jaw tumors, the patient was admitted for jaw debulking surgery followed by histopathological correlation. Total parathyroidectomy was also planned. However, the patient’s condition and her renal function status worsened. Urgent dialysis was planned in the ICU and a central venous catheter was placed. After a single session of dialysis, she developed multiorgan failure. Despite aggressive management, her condition deteriorated and she did not survive. The patient’s family members initially refused her genetic testing due to unaffordability but they were counseled for the genetic testing of other members of the family.

## Discussion

Fibro-osseous lesions of HPT-JT syndrome most commonly involve the mandible and maxilla. Osseous involvement is seen in 30% to 40% of the cases [6]. The majority of the HPT-JT syndrome-related osseous tumors reported in the literature are benign, slow-growing ossifying fibromas arising from molar or premolar, periodontal ligament and mostly presenting as enlarging palpable or visible radiolucent mass lesions. Sporadic cases present as mixed lytic sclerotic lesions [5]. Jaw tumors resemble ossifying fibromas in histopathological respect with the presence of giant cells. Major imaging differentials include fibrous dysplasia and osteolytic brown tumors of hyperparathyroidism. However fibrous dysplasia exhibits ill-defined expansile bony lesions, while Brown tumors present with purely lytic lesions without any sclerotic border. In the light of the fact that many benign osseous-fibrous lesions share overlapping microscopic features, a clinical-radiological correlation is often required for their diagnosis [7].

The youngest age at presentation for HPT-JT syndrome reported in the literature is 10 years [8]. In a study conducted by Le Collen et al., a family carrier of CDC73 gene exon 3 deletion was reported characterized by hypercalcemia, renal deterioration, and atypical parathyroid adenomas [9].

Though of benign etiology, ossifying fibromas cause significant local tissue destruction. This can lead to cosmetic disfigurement and functional disability in the form of breathing difficulties, periorbital swelling, and abnormal dentition. The risk of malignant degeneration is less than 0.5%. Considering the multifocality and higher risk of recurrence, complete surgical removal followed by bone grafting and reconstruction is the treatment of choice. Such patients must be followed closely to rule out recurrence of jaw tumors and associated higher risk of development of parathyroid tumors [10].

Oral and dental surgeons must be aware of the possibility of HPT-JT syndrome in adolescents and young adults who present with a jaw mass and high serum PTH levels, either as an independent clinical entity or in conjunction. Keeping in view the patient’s clinical presentation, here comes the role of detailed probing of the patient’s family history and genetic analysis for CDC73 genetic mutation [8].

Renal manifestations account for 15% of HPT-JT syndrome patients with cystic renal disease being the most common pathology encountered. Less common renal diseases include hamartomas, Wilms tumor, and mixed epithelial-stromal tumors (MEST). Wilms tumor presents in the fifth decade of life, being smaller in size than childhood form. The risk of renal tumors in HPT-JT syndrome patients predisposes them to multiple renal surgeries. In order to salvage renal function, nephron-sparing surgery is preferred over radical nephrectomy [4].

Surgery remains the mainstay of treatment for the most common manifestation of HPT-JT syndromes such as primary hyperparathyroidism secondary to parathyroid adenoma or carcinoma [11]. Limited parathyroid excision is preferred over total parathyroidectomy to reduce the risk of hypoparathyroidism and significant morbidity. In patients who are not potential candidates for surgery, Cinacalcet has proved its efficacy in alleviating symptoms of primary hyperparathyroidism. Patients should however be informed of a post-surgical disease recurrence risk of 25% [5]. Genetic testing should be considered in early-onset hyperparathyroidism patients in order to adopt appropriate surgical management protocol depending on the life expectancy of the patient [12].

Gynecological and obstetric manifestations also require surgical management mainly in the form of hysterectomy in adequate clinical settings. Women of the reproductive age group should maintain normal serum calcium levels before conception to prevent maternal and fetal complications [5].

In a nutshell, surgical management is the most appropriate treatment approach for the pathologies encountered in HPT-JT syndrome. Genetic testing remains the gold standard for diagnosis, with screening advisable for patients presenting with reduced serum parathormone levels. Taking into consideration that there is a 25% recurrence risk of HPT-JT syndrome-related tumors, long-term follow-up is indicated in all the patients [3].

## Conclusions

This case report aims to promote awareness regarding the rare pathologic condition of HPT-JT syndrome and its various clinical associations resulting in morbidity, facial disfigurement, and malignancy. According to the literature review, we conclude that although no formal guidelines are available for the management of this infrequent diagnosis of HPT-JT syndrome. However, all CDC73 mutation carriers should be followed up. This can include biochemical evaluation of PTH and serum calcium levels at least twice a year starting from the age of five years, neck ultrasound for parathyroid gland assessment, five-yearly orthopantomogram (OPG), five-yearly renal examination by ultrasound, CT, or MRI, and regular pelvic sonographic evaluation in gynecological respect starting from the reproductive age. Attending physicians must keep a low threshold for genetic testing in primary hyperparathyroid patients by extending genetic testing to their offspring as well as relatives. This topic is of immense importance for the radiologists who must keep a high index of suspicion for HPT-JT syndrome in patients presenting with a jaw malignancy. In our case, clinical, biochemical, and radiological correlation supports the aforementioned diagnosis.
